# Carotid Anatomy Does Not Predict the Risk of New Ischaemic Brain Lesions on Diffusion-Weighted Imaging after Carotid Artery Stenting in the ICSS-MRI Substudy

**DOI:** 10.1016/j.ejvs.2015.08.012

**Published:** 2016-01

**Authors:** D. Doig, B.M. Hobson, M. Müller, H.R. Jäger, R.L. Featherstone, M.M. Brown, L.H. Bonati, T. Richards, the ICSS-MRI Study Investigators

**Affiliations:** a Institute of Neurology, University College London, UK; b University College London Medical School, UK; c University of Basel, Basel, Switzerland; d Department of Neurology and Stroke Unit, University Hospital Basel, Basel, Switzerland; e Division of Surgery and Interventional Science, University College London, UK

**Keywords:** Carotid atherosclerosis, Carotid artery stenosis, Carotid artery ulcerating plaque, Stents, Brain ischaemia

## Abstract

**Introduction:**

The International Carotid Stenting Study (ICSS, ISRCTN25337470) randomized patients with recently symptomatic carotid artery stenosis > 50% to carotid artery stenting (CAS) or endarterectomy. CAS increased the risk of new brain lesions visible on diffusion-weighted magnetic resonance imaging (DWI-MRI) more than endarterectomy in the ICSS-MRI Substudy. The predictors of new post-stenting DWI lesions were assessed in these patients.

**Methods:**

ICSS-MRI Substudy patients allocated to CAS were studied. Baseline or pre-stenting catheter angiograms were rated to determine carotid anatomy. Baseline patient demographics and the influence of plaque length, plaque morphology, internal carotid angulation, and external or common carotid atheroma were examined in negative binomial regression models.

**Results:**

A total of 115 patients (70% male, average age 70.4) were included; 50.4% had at least one new DWI-MRI-positive lesion following CAS. Independent risk factors increasing the number of new lesions were a left-sided stenosis (incidence risk ratio [IRR] 1.59, 95% CI 1.04–2.44, *p* = .03), age (IRR 2.10 per 10-year increase in age, 95% CI 1.61–2.74, *p* < .01), male sex (IRR 2.83, 95% CI 1.72–4.67, *p* < .01), hypertension (IRR 2.04, 95% CI 1.25–3.33, *p* < .01) and absence of cardiac failure (IRR 6.58, 95% CI 1.23–35.07, *p* = .03). None of the carotid anatomical features significantly influenced the number of post-procedure lesions.

**Conclusion:**

Carotid anatomy seen on pre-stenting catheter angiography did not predict of the number of ischaemic brain lesions following CAS.

**What this paper adds**

The morphology, angulation and degree of carotid stenosis in patients undergoing CAS did not predict the occurrence of new DWI-positive brain lesions. Patients should not be excluded from CAS based on their carotid anatomy.

## Introduction

Carotid artery stenting (CAS) is an endovascular alternative to carotid endarterectomy (CEA) for the long-term prevention of recurrent stroke in patients with carotid atheroma. CAS was tested against CEA in the International Carotid Stenting Study (ICSS), a large randomized controlled trial recruiting symptomatic patients with greater than 50% carotid artery stenosis. Interim analysis of this trial demonstrated an excess of stroke, death, or myocardial infarction in patients randomized to CAS, mostly attributable to an excess of minor stroke.^1^ This observation was reinforced in the ICSS-MRI Substudy, which found a 50% incidence of new brain lesions in patients receiving CAS treatment in the trial compared with just 17% of patients receiving CEA (OR 5.21, 95% CI 2.78–9.79, *p* < .01).^2^ Patients in the CAS group had a greater number of lesions, and these lesions were more likely to occur in cortical and in adjacent white matter areas.^3^

CAS requires the passage of devices through the vasculature up to or beyond the stenotic lesion. Vascular anatomical variants and atheromatous lesion characteristics have therefore been proposed as contributing to a technically challenging procedure^4^ or a higher burden of ischaemic brain lesions post procedure.^5^ The study aimed to investigate the effects of carotid vascular anatomy and plaque morphology on the occurrence of new ischaemic brain lesions on diffusion-weighted magnetic resonance imaging (DWI-MRI) following CAS, and whether there are patient or procedural factors that place patients at higher risk of this complication.

## Methods

### ICSS-MRI substudy

The ICSS-MRI Substudy was contained within the International Carotid Stenting Study, which randomized patients to either CAS or CEA. Full inclusion and exclusion criteria are published in the ICSS protocol.^6^ The clinical short-term and long-term outcomes have been previously published.^1^, ^7^ Centres participating in the ICSS-MRI substudy are published elsewhere.^2^ In summary, patients older than 40 years were required to have > 50% recently symptomatic carotid artery stenosis, be suitable for either surgery or stenting, and be capable of giving informed consent. Patients were excluded from randomization in ICSS if CAS was judged unsuitable because of pseudo-occlusion of the artery (stenosis ≥ 95% with collapsed distal lumen), tortuous vascular anatomy proximal or distal to the stenosis, proximal common carotid artery stenosis, or plaque thrombus visible on imaging. CAS was carried out by accredited interventionists according to a strict protocol.^6^ A cerebral protection device (CPD) was recommended where one could be “safely deployed”. Dual antiplatelet therapy to cover the procedure and for a minimum of 4 weeks thereafter was specified in the protocol.

Patients in the MRI substudy underwent pre-procedural MRI up to 7 days before CAS. Post-procedure MRI was performed 1–3 days after CAS. All imaging protocols included diffusion-weighted imaging sequences to demonstrate areas of acute or subacute brain ischaemia or infarction. DWI lesion count was determined by the consensus reading of a neurologist and neuroradiologist blind to treatment allocation.^2^, ^3^ Age-related white matter changes (ARWMC) on brain imaging were rated by two investigators as proposed by Wahlund and colleagues.^8^, ^9^

Patients in this analysis were those in the ICSS-MRI substudy in whom the allocated procedure of CAS was initiated and a baseline or pre-stenting intra-arterial catheter angiogram was available. Baseline patient characteristics were recorded by investigators at each centre at the time of the patient's enrolment in the study. These included the side of the stenosis, vascular risk factors such as baseline blood pressure and cholesterol, and whether hypertension or hypercholesterolaemia were treated, a diagnosis of diabetes, heart failure, peripheral vascular disease or cardiac failure, and baseline disability as measured by the modified Rankin scale (mRS).

ICSS was approved by the Northwest Multicentre Research Ethics Committee in the UK. Individual participating centres obtained site-specific approval from their local ethics committees.

### Anatomical rating methods

Baseline catheter angiography, performed at the time of enrolment into the trial or procedural angiography prior to stent deployment was evaluated by a trained reader (B.M.H.). A second trained reader (D.D.) made comparator measurements to enable inter-rater reliability to be defined. Both readers were blind to our selected outcome of lesion count at the time of reading. Standard definitions were developed for rating anatomies. The degree of stenosis in the ipsilateral treated artery was calculated using the NASCET formula^10^: the narrowest residual lumen compared with the distal ICA diameter. Ulceration or plaque irregularity was defined in accordance with NASCET plaque morphology rating, being ulcerated when “seen in profile as a crater penetrating into a stenotic plaque”.^11^ The angle between the common carotid artery (CCA) and internal carotid artery (ICA) was calculated. “Pinhole” stenosis was defined as an arterial lumen too small to accurately measure but with normal distal runoff. “Length of stenosis” was defined as the distance between proximal and distal shoulders of the lesion where the luminal diameter decreased to 20% of its maximum, expressed as a fraction of the CCA diameter on angiography views that most elongated that stenosis.^12^ The CCA was judged to be “diseased” with atheroma if there was definite plaque encroaching on the lumen or irregularity in the lumen consistent with atherosclerosis. Views of the contralateral carotid artery were not routinely available; therefore the measurement of contralateral stenosis provided by the local investigator at the point of entry into the trial was used. DICOM images were read using the open-source software Osirix MD, Pixmeo SARL (2012).

### Statistical methods

Negative binomial regression models were constructed for each risk factor investigated. Comparisons between a reference group and the factor under consideration are expressed as incidence risk ratios (IRRs) with a 95% Wald confidence interval, quantifying how many times more or fewer lesions occurred. Factors with *p* < .05, considered significant in all analyses, were considered for entry into a multivariable model. Inter-rater agreement for vascular anatomical variables was assessed by means of a kappa (κ) statistic as proposed by Landis and Koch.^13^ Statistical analyses were performed using SPSS version 22.0.0.0, IBM Corp (Armonk, NY, USA).

## Results

### Baseline patient and procedural characteristics

A total of 231 patients were randomized in the ICSS-MRI substudy, of whom 127 were randomized to CAS. Baseline (*n* = 7) or pre-procedure (*n* = 110) catheter angiography, with views adequate to assess anatomical characteristics, was available for 115 patients, all of whom were included in this analysis (2 patients had both baseline and pre-procedure imaging). The mean age was 70.4 years; 70% of patients were male. Vascular risk factors were common, as detailed in Table 1. Forty-seven per cent of procedures were carried out on the left carotid, 53% on the right. A protection device was used in 46 out of 115 (40.0%) procedures, of which 91% were distal filter type and 9% were flow reversal systems. Overall, the median carotid stenosis was 62.7% (IQR 52.0–78.9). Plaque morphology showed ulceration in 40 patients (35%). Angulation and external carotid artery (ECA) disease were less common. A quarter (30, 26%) had severe contralateral carotid artery disease. Following CAS 62/115 (50.4%) of patients had at least one new DWI-MRI-positive lesion on their post-procedure scan. Details of the distribution of lesion count can be found in Gensicke et al.^3^

### Univariable results

Table 2 summarizes the results of analysis of patient demographic, vascular anatomical characteristics and procedural factors in a negative binomial regression analysis. The number of new DWI-MRI lesions at 1–3 days after the CAS procedure was associated with increasing age (IRR 2.26 for each 10-year increase in age, 95% CI 1.74–2.89, *p* < .01), hypertension (IRR 2.61, 95% CI 1.68–4.05, *p* < .01), with increasing ARWMC (IRR 1.13 for each point increase, 95% CI 1.06–1.20, *p* < .01), and with stroke or transient ischaemic attack (TIA) as the qualifying event for entry into ICSS, compared with those with a retinal event (IRR 4.30, 95% CI 2.46–7.53, *p* < .01 for stroke, IRR 1.91, 95% CI 1.06–3.43, *p* = .03 for TIA). The number of new DWI lesions was significantly lower in female patients (IRR 0.49, 95% CI 0.31–0.76, *p* < .01) and current or former smokers than in those who had never smoked (IRR 0.48, 95% CI 0.31–0.76, *p* < .01), those with cardiac failure (IRR 0.17, 95% CI 0.03–0.85, *p* = .03), those with 50–69% contralateral carotid stenosis than in those with < 50% contralateral carotid stenosis (IRR 0.18, 95% CI 0.08–0.40, *p* < .01) and in those with a modified Rankin scale score (mRS) of 3 at baseline compared to those with an mRS score of 0 (IRR 0.20, 95% CI 0.06–0.62, *p* < .01). None of the anatomical variables (degree and length of ipsilateral stenosis, the CCA to ICA angle, ECA stenosis, carotid ulceration, or the presence of CCA atheroma) measured on ipsilateral pre-procedure angiography predicted the occurrence of new DWI-MRI lesions post procedure. However, right-sided stenosis was associated with a lower rate of new lesions versus left-sided stenosis.

### Multivariable model

Factors with significance *p* < .05 were considered for entry into a multivariable model. After adjustment for other characteristics, the type of qualifying event, smoking status, and baseline disability were no longer significant predictors. Table 3 illustrates one possible multivariable model with five simple clinical characteristics – the side of the procedure, age, sex, hypertension, and cardiac failure. Independent risk factors increasing the number of new DWI-MRI lesions following CAS were age (IRR 2.10 per 10-year increase in age, 95% CI 1.61–2.74, *p* < .01), hypertension (IRR 2.04, 95% CI 1.25–3.33, *p* < .01), a left-sided stenosis (IRR 1.59, 95% CI 1.04–2.44, *p* = .03), male sex (IRR 2.83, 95% CI 1.72–4.67, *p* < .01), and an absence of cardiac failure (IRR 6.58, 95% CI 1.23–35.07, *p* = .03).

### Inter-rater reliability

Inter-rater agreement was moderate for CCA atheroma (κ = 0.50, *p* < .01), substantial for the type of stenosis (smooth, irregular or ulcerated, κ = 0.76, *p* < .01), and slight for pinhole stenosis (κ = 0.13, *p* = .06).

## Discussion

In this study of patients in the ICSS-MRI Substudy, anatomy of the carotid artery or plaque morphology was not found to predict the number of new post-procedure DWI-MRI lesions following intervention by CAS. Consequently, the increased event rate following CAS could not be explained by inclusion of patients with higher risk carotid anatomy. Expert opinion has proposed that certain vascular anatomies render CAS more difficult, such as vessel angulation or tortuosity or aortic arch variants.^4^ Other authors have determined a number of variables that could specifically affect the risk of new DWI brain lesions following CAS, including age,^5^ an ulcerated stenosis,^5^ aortic atherosclerotic lesions,^14^ and increasing lesion length.^5^ Studies measuring clinical outcomes of stroke or peri-procedural stroke, death, or myocardial infarction have suggested lesion characteristics such as length,^12^, ^15^, ^16^ plaque ulceration or calcification,^15^ aortic arch anatomy,^15^, ^17^ ICA–CCA angulation,^16^ and the side of the procedure^16^ influence the risk of complications. In this analysis, a higher number of lesions were noted in left-sided procedures, perhaps reflecting increased difficulty in access to the CCA on this side.^16^ However, the findings of others regarding the influence of pinhole stenosis, length of stenosis, or ICA–CCA angulation on procedural risk could not be replicated.

At most centres in ICSS the anatomical suitability of the stenosis for CAS and CEA was determined prior to randomization using non-invasive imaging such as ultrasound, computed tomography (CT) angiography or magnetic resonance (MR) angiography. Pre-stenting catheter angiograms were primarily obtained to plan the procedures; the films available for analysis usually did not include the aortic arch and did not allow assessment of all angles in the CCA and ICA. Another possible explanation for the lack of an association between DWI lesions and anatomical parameters is that patients thought to be at high risk from stenting were excluded from taking part in ICSS. Indeed, the trial protocol specifically excluded patients with a stenosis that was known to be unsuitable for stenting prior to randomization.^6^ Thus a relatively low percentage of patients with pinhole stenosis or angulation of the CCA–ICA junction are reported. We also note low inter-rater reliability in other studies for some characteristics measured^11^ in agreement with the results of this study. This represents a major limitation if vascular characteristics are proposed for patient selection: there should be reliable agreement as to which patients may be at increased risk. In the future, access to advanced plaque imaging, perhaps with MRI, could increase the accuracy and reliability of pre-procedure plaque characterization by examining plaque characteristics and morphology.

Older patients experienced a higher number of new ischaemic brain lesions following CAS in the ICSS-MRI Substudy, and an association between the total volume of DWI lesions and hemispheric stroke in this patient group has previously been demonstrated.^2^ This finding echoes clinical data from the Carotid Stenting Trialists' Collaboration, which conducted an individual patient data meta-analysis of the large European trials of CAS versus CEA, namely ICSS,^1^ EVA-3S,^18^ and SPACE.^19^ This found a twofold increase in the risk of stroke, death, or myocardial infarction within 30 days of CAS compared with CEA in patients over the age of 70 years.^20^ The mechanism by which age increases risk is not apparent from this study, but may be related to an underlying increase in the burden of atheroma over time or a decreased ability to tolerate reduced blood flow in the brain after embolism. Changes in aortic arch anatomy and atherosclerotic burden with age may also explain concerns about the formation of new DWI lesions following cardiac catheterization where the carotid artery is not catheterized.^21^ Male patients and those with hypertension also experienced an increased number of post-procedure DWI lesions. Although it is difficult to postulate a direct causal mechanism for this, unmeasured factors in these patients, such as the type of plaque (haemorrhagic, lipid-rich) or the total plaque burden, may have made a greater contribution to inadvertent embolization during the procedure. Large-scale registries, such as that of Medicare patients in the United States undergoing CAS, suggest a similar risk of minor stroke within 30 days of CAS in men and women,^22^ and therefore the excess of ischaemic lesions in men in our study may be subclinical. Other unmeasured variables may confound the findings with respect to cardiac failure; for example, it is possible that patients with this diagnosis received more appropriate antiplatelet cover for the procedure than others.

The finding that CPD use did not protect against new DWI lesions agrees with two small randomized studies of protected versus unprotected CAS which used MRI up to 24 hours after the procedure to look for evidence of peri-procedural cerebral ischaemia.^23^, ^24^ Some studies report that CPDs do reduce emboli, but even these analyses show that DWI lesions still frequently occur following protected CAS.^25^, ^26^ Most CPDs used in ICSS were distal filters, and research continues to determine whether newer flow-reversal types of CPD may confer better protection.^27^

### Limitations

This analysis has a number of important limitations. Views on catheter angiography were limited, and it was not possible to evaluate the anatomy of the intracranial portion of the ICA, the proximal CCA, or the aortic arch, all of which may be important areas of technical difficulty in the CAS procedure. It should be noted that CPD use in ICSS was recommended when safe, but still at the interventionist's discretion. Thus the comparison between patients with CPD or no CPD is not a randomized comparison. Anatomical analysis was performed on catheter angiography, but in current practice selection of patients for CAS is likely to be based on CT angiography or MR angiography. However, it is expected that many of the features that were identified, including pinhole stenosis, ICA angulation, and plaque length, would correlate well with the equivalent measurements on non-invasive imaging. No corrections were made for multiple statistical comparisons in univariable analysis, given the low number of patients with an outcome event (*n* = 62) and the limited power to detect a true difference, giving rise to the possibility of type I (false positive) errors in interpretation.

## Conclusion

In patients otherwise matching the inclusion and exclusion criteria for ICSS, there is no reason to exclude patients from stenting on the basis of individual vascular anatomical characteristics measured in the analysis. However, patient characteristics such as age and sex should be taken into account when selecting for CAS.

## Conflict of Interest

None.

## Funding

ICSS was funded by the UK Medical Research Council (MRC, grant number G0300411) and managed by National Institute for Health Research (NIHR) NIHR on behalf of the MRC-NIHR partnership. Additional funding was received from the Stroke Association (grant numbers TSA 2005/01 and TSA 2007/12), Sanofi-Synthélabo and the European Union. The ICSS-MRI Substudy received funding from the Mach-Gaensslen Foundation in Switzerland, The Netherlands Heart Foundation and the Stroke Association in the UK. L.B. is supported by grants from the Swiss National Science Foundation (Grant number PBBSB-116873) and the University of Basel. M.M.B.'s chair in stroke medicine is supported by the Reta Lila Weston Trust for Medical Research. D.D. and R.L.F. were supported by grants from the MRC. This work was partly performed at University College London Hospital and University College London, which received a proportion of funding from the Department of Health National Institute for Health Research Biomedical Research Centres funding scheme. The views and opinions expressed therein are those of the authors and do not necessarily reflect those of the NIHR HSR programme or the Department of Health. The funders of this study had no role in the design of the trial or this analysis and did not contribute to the manuscript.

## Figures and Tables

**Table 1:** Baseline and measured characteristics of patients included in the analysis.

Parameter	Number of patients (%) total *n* = 115
**Demographic and technical**	
Right-sided stenosis	61 (53%)
Cerebral protection device used	46 (40%)
Age, years (mean, standard deviation)	70.4 (SD = 9.50)
Female	34 (30%)
Hypertension	77 (67%)
Diabetes (any type)	22 (19%)
Treated hyperlipidaemia	71 (62%)
Current or former smoker	87 (76%)
Coronary heart disease	26 (23%)
Other cardioembolic source of thrombus	7 (6%)
Cardiac failure	3 (3%)
Peripheral arterial disease	21 (18%)
Contralateral carotid occlusion	7 (6%)
Contralateral carotid stenosis 70–99%	23 (20%)
Contralateral carotid stenosis 50–69%	12 (10%)
Contralateral carotid stenosis < 50% (reference group)	73 (64%)
Baseline modified Rankin Scale score (mRS) = 4	2 (2%)
Baseline mRS = 3	6 (5%)
Baseline mRS = 2	28 (24%)
Baseline mRS = 1	29 (25%)
Baseline mRS = 0 (reference group)	50 (44%)
Cholesterol (mean, standard deviation)	4.9 mmol/L (SD = 1.34)
Baseline diastolic blood pressure (mean, standard deviation)	83.0 mmHg (SD = 12.7)
Baseline systolic blood pressure (mean, standard deviation)	157.3 mmHg (SD = 26.1)
Median age-related white matter change score (ARWMC)	4
Qualifying event was stroke	49 (43%)
Qualifying event was transient ischaemic attack (TIA)	41 (36%)
Qualifying event was retinal TIA or retinal stroke	25 (22%)
**Vascular anatomical characteristics**	
Carotid artery NASCET degree of stenosis (median, interquartile range)	62.7 (IQR 52.0–78.9)
Ratio of maximal stenosis length to common carotid artery diameter (median, interquartile range)	0.41 (IQR 0.26–0.65)
Angle between common and internal carotid arteries (median, interquartile range)	23.6° (IQR 15.6–35.9)
External carotid artery stenosis >50%	6 (5%)
Plaque ulceration	40 (35%)
Pinhole stenosis	2 (2%)
Common carotid artery atheroma	13 (11%)

**Table 2:** Univariable predictors of new DWI-MRI lesions following CAS in 115 patients.

Parameter	*p*	Incidence Risk Ratio (IRR)	95% Confidence interval for IRR	
Lower	Upper
**Demographic and technical**				
Right-sided stenosis	< .01	0.57	0.38	0.85
Cerebral protection device used	.22	1.30	0.86	1.95
Age (per 10-year increase)	< .01	2.26	1.74	2.89
Female	< .01	0.49	0.31	0.76
Hypertension	< .01	2.61	1.68	4.05
Diabetes (any type)	.07	1.59	0.97	2.61
Treated hyperlipidaemia	.69	0.92	0.61	1.38
Current or former smoker	< .01	0.48	0.31	0.76
Coronary heart disease	.43	1.21	0.76	1.94
Other cardioembolic source of thrombus	.28	1.56	0.69	3.49
Cardiac failure	.03	0.17	0.03	0.85
Peripheral arterial disease	.29	0.75	0.45	1.27
Contralateral carotid occlusion	.21	1.69	0.75	3.80
Contralateral carotid stenosis 70–99%	.12	0.66	0.39	1.11
Contralateral carotid stenosis 50–69%	< .01	0.18	0.08	0.40
Contralateral carotid stenosis < 50% (reference group)	N/A	1	N/A	N/A
Baseline modified Rankin Scale score (mRS) = 4	.14	0.26	0.04	1.57
Baseline mRS = 3	< .01	0.20	0.06	0.62
Baseline mRS = 2	.20	1.38	0.84	2.26
Baseline mRS = 1	.40	0.81	0.49	1.33
Baseline mRS = 0 (reference group)	N/A	1	N/A	N/A
Cholesterol (per 1 mmol increase in serum total cholesterol)	.30	0.89	0.71	1.11
Baseline diastolic blood pressure (per 10 mmHg increase)	.13	0.90	0.79	1.03
Baseline systolic blood pressure (per 10 mmHg increase)	.98	1.00	0.91	1.09
Age-related white matter changes score (per unit increase)	< .01	1.13	1.06	1.20
Qualifying event was stroke	< .01	4.30	2.46	7.53
Qualifying event was transient ischaemic attack (TIA)	.03	1.91	1.06	3.43
Qualifying event was retinal TIA or retinal stroke (reference group)	N/A	1	N/A	N/A
**Vascular anatomical characteristics**				
Carotid artery NASCET degree of stenosis (per 10% increase)	.69	0.97	0.83	1.13
Ratio of maximal stenosis length to common carotid artery diameter (per unit increase)	.46	1.28	0.67	2.45
Angle between common and internal carotid arteries	.12	0.99	0.98	1.00
External carotid artery stenosis > 50%	.08	0.42	0.16	1.10
Plaque ulceration	.66	1.10	0.73	1.66
Pinhole stenosis	.88	1.13	0.25	5.05
Common carotid artery atheroma	.69	0.88	0.47	1.65

**Table 3:** Independent predictors of new DWI-MRI lesions following CAS in 115 patients.

Parameter	*p*	Incidence Risk Ratio (IRR)	95% Confidence interval for IRR	
Lower	Upper
Left-sided stenosis	.03	1.59	1.04	2.44
Age (per 10-year increase)	< .01	2.10	1.61	2.74
Male	< .01	2.83	1.72	4.67
Hypertension	< .01	2.04	1.25	3.33
No diagnosis of cardiac failure	.03	6.58	1.23	35.07
